# Genomic and Physiological Properties of a Facultative Methane-Oxidizing Bacterial Strain of *Methylocystis* sp. from a Wetland

**DOI:** 10.3390/microorganisms8111719

**Published:** 2020-11-02

**Authors:** Gi-Yong Jung, Sung-Keun Rhee, Young-Soo Han, So-Jeong Kim

**Affiliations:** 1 Geologic Environment Research Division, Korea Institute of Geoscience and Mineral Resources, Daejeon 34132, Korea; seraphim0123@gmail.com; 2 Department of Microbiology, Chungbuk National University, Cheongju 28644, Korea; rhees@chungbuk.ac.kr; 3 Department of Environmental Engineering, Chungnam National University, Daejeon 34134, Korea; hanyoungsoo@cnu.ac.kr

**Keywords:** methanotrophic bacteria, genome of *Methylocystis*, wetland, methane monooxygenase

## Abstract

Methane-oxidizing bacteria are crucial players in controlling methane emissions. This study aimed to isolate and characterize a novel wetland methanotroph to reveal its role in the wetland environment based on genomic information. Based on phylogenomic analysis, the isolated strain, designated as B8, is a novel species in the genus *Methylocystis*. Strain B8 grew in a temperature range of 15 °C to 37 °C (optimum 30–35 °C) and a pH range of 6.5 to 10 (optimum 8.5–9). Methane, methanol, and acetate were used as carbon sources. Hydrogen was produced under oxygen-limited conditions. The assembled genome comprised of 3.39 Mbp and 59.9 mol% G + C content. The genome contained two types of particulate methane monooxygenases (pMMO) for low-affinity methane oxidation (pMMO1) and high-affinity methane oxidation (pMMO2). It was revealed that strain B8 might survive atmospheric methane concentration. Furthermore, the genome had various genes for hydrogenase, nitrogen fixation, polyhydroxybutyrate synthesis, and heavy metal resistance. This metabolic versatility of strain B8 might enable its survival in wetland environments.

## 1. Introduction

Wetlands, including bogs, marshes, and swamps, are typical areas where the methane cycle occurs actively and play a role in the carbon cycle [1,2]. Owing to methane production by methanogens, eutrophic wetlands are the main channel of methane fluxes [3,4]. Methane diffuses throughout wetlands [5,6] and creates a suitable environment for methanotrophs. Methanotrophic metabolism contributes to carbon cycling in wetland ecosystems [7,8]. The emission of methane is a major contributor to the greenhouse effect [9]. The importance of methanotrophic microorganisms in wetland carbon cycling and methane emissions has spurred investigations to identify and characterize aerobic methanotrophs. 

Type I (including a subdivision of type X) methanotrophs belong to the class *Gammaproteobacteria* and Type II methanotrophs belong to the class *Alphaproteobacteria* [10,11]. Methanotrophs belonging to *Verrucomicrobia* were discovered in acidic thermal environments. Methane oxidation by methane monooxygenase is the first and most critical step in methane metabolism [12,13,14]. Methane is oxidized by particulate methane monooxygenase (pMMO) and soluble methane monooxygenase (sMMO) [15]. Whereas pMMO is bound to the cell membrane, sMMO exists in the cytoplasm [15]. Both pMMO and sMMO require distinct metal elements as a prosthetic group (copper ions and iron ions, respectively) [16,17,18]. Methanol is oxidized to formaldehyde by methanol dehydrogenase [19]. Formaldehyde is oxidized by several enzymes, including the formaldehyde-activating enzyme, through the methylene tetrahydromethanopterin (H_4_MPT) pathway [20,21]. Formate is oxidized by formate dehydrogenase to supply electrons to NAD [22]. Formaldehyde and formate are used as carbon sources depending on the methanotroph. In the RuMP pathway of type I methanotrophs, formaldehyde is used for cellular carbon production. Formaldehyde and ribulose 5-phosphate are converted to hexulose 6-phosphate through the pentose phosphate pathway, which is used to synthesize cellular carbohydrates. In the serine pathway of type II methanotrophs, formate is converted to methylenetetrahydrofolate (THF) and used for carbon metabolism [22].

Methanotrophs are not only able to reduce methane but can also produce value-added substances [23]. Recently, methanotrophs have become a promising option for mitigating methane emission [24]. *Methylocystis parvus* OBBP was known as one of the candidates for the cell factory production of Polyhydroxybutyrate (PHB) [25]. Other studies have been conducted on the synthesis of PHB by methane-oxidizing bacteria [26]. The production of organic acids (formate, acetate, lactate, and succinate) and hydrogen in *Methylomicrobium alcaliphilum* 20Z was considered a potential biocatalysis based on methane [27]. 

The genus *Methylocystis* includes type II methanotrophs belonging to the class *Alphaproteobacteria* and family *Methylocystaceae* [28]. The genus *Methylocystis* was first identified in 1970 [29] and was later validated by Bowman et al. [28]. The genus *Methylocystis* includes six species: *M. rosea* [30], *M. hirsuta* [31], *M. bryophila* [32], *M. echinoides*, *M. parvus* [28], and *M. heyeri* [33]. These species have been isolated from various environments, including groundwater aquifers, acidic peat-bog lakes, and wetland soils. Several *Methylocystis* genomes have been reported [34,35,36,37,38]. 

Methanotrophs were initially known as microorganisms which used only methane and methanol as carbon and energy sources. However, starting with *Methylocella palustris*, among the verified strains, it has been reported that facultative methanotrophs could utilize compounds with carbon-carbon bonds. The genus *Methylocystis* included some facultative methanotrophic species. The common source of multi-carbon was acetate. A study assumed that acetate utilization might be important for survival in environments where methane availability is variable or limited [39]. Such cases included wetlands and upland soils. *Methylocystis* spp. were predominant in these environments [40,41].

In this study, a novel methanotroph was isolated from a wetland and characterized to reveal its role in methane cycling in this environment. Genome properties were revealed using comparative genomics. The physiological and genomic data provide insight into the adaptations of *Methylocystis* methanotrophs in wetland environments.

## 2. Materials and Methods

### 2.1. Enrichment Culture and Isolation

A water sample was obtained from an artificial wetland in Ok-Cheon, Republic of Korea (36.19 N, 127.33 E) in June 2017. Temperature, electrical conductivity (EC), oxidation-reduction potential (ORP), pH, and dissolved oxygen (DO) of sampled water were checked by a Multimeter (HQ40d, Hach, Loveland, CO, USA) onsite. To examine the water composition, the water was filtered with a syringe filter (0.45 μm pore size) and analyzed with ion chromatography (IC, Metrohm, Herisau, Switzerland) and inductively coupled plasma-optical emission spectrometry (ICP-OES, Horiba Jobin Yvon, Longjumeau, France). The 50 mL sample was filtered through a 0.1 μm pore polycarbonate (PC) filter membrane to collect cells. The collected cells were incubated in 10 mL of modified nitrate mineral salt (NMS) medium in a Balch-type tube (CLS-4209, Chemglass, Vineland, NJ, USA) at 25 °C. Methane (filter sterilized, 0.22 μm pore size) was added to the headspace air (15%, *v/v*) through a syringe. One liter of the medium contained 0.0985 g MgSO_4_·7H_2_O, 0.0147 g CaCl_2_·2H_2_O, 0.0348 g K_2_SO_4_, 0.017 g NaNO_3_, 0.0136 g KH_2_PO_4_, 0.252021 g NaHCO_3_, 1 mL SL-10 trace element solution [42], 1 mL vitamin solution [43], and 0.028 g cerium [44]. The culture was serially diluted from 10^−1^ to 10^−10^ several times. Furthermore, the dilution-to-extinction cultivation method was conducted in 96-well plates. The emerging turbidity among the wells was used for isolation using the floating membrane method with the polycarbonate membrane (0.1 μm pore size) on the NMS medium at various methanol concentrations (0.1%, 0.5%, and 1%) at 25 °C. After 2 weeks, small colonies were observed on the floating membrane. The isolated cell was named B8. Cells were routinely cultured in a modified NMS medium. The purity of culture was checked by monitoring the T-RFLP of 16S rRNA gene PCR product [45] from cultures grown with either methane or acetate after incubation. In addition, no growth was confirmed in the modified NMS medium with 0.1% yeast extract as the sole energy source. Furthermore, contamination by other heterotrophic bacteria was routinely checked on nutrient agar and R2A agar plates.

### 2.2. Physiologic Analysis

Gram staining was performed using a commercial kit (BD, Franklin Lakes, NJ, USA). Cell morphology and cell size were observed by differential interference contrast microscopy (Axio Scope.A1; Carl Zeiss, Jena, Germany). Catalase and oxidase tests were performed with 3% hydrogen peroxide (H_2_O_2_) and oxidase strips (Sigma-Aldrich. St. Louis, MO, USA), respectively. Strain B8 was grown at 4 °C, 10 °C, 15 °C, 20 °C, 25 °C, 30 °C, 35 °C, 37 °C, 40 °C, and 45 °C. NaCl tolerance was assessed by growth in the presence of 0% to 5% NaCl (in 0.5% increments). The pH was tested from 4 to 12 in increments of 0.5. To identify nitrogen sources used by strain B8, the sample was incubated in a modified NMS medium with various nitrogen sources (cysteine, yeast extract, casamino acid, betaine, carsitone, and ammonium sulfate) by replacing 0.05% (*w/v*) sodium nitrate. Carbon source utilization was assessed using 0.05% ethanol, glucose, acetate, or pyruvate. Tests were performed in duplicates. Colony formation was checked on the solid medium containing 1.5% agarose, 1% phytagel, and 1% gellan gum. PHB formation was estimated using Nile Red staining followed by examination using an Axio Scope A1 fluorescence microscope (Carl Zeiss, Oberkochen, Germany). To calculate the growth rate, strain B8 was cultured in a modified NMS medium with added methane (15%, *v/v*) at 30 °C and 150 rpm. The growth rate was monitored in triplicates. The methane concentration in headspace and cell growth was measured for 7 days. Cell growth (optical density at 600 nm, OD_600_) was determined using spectrophotometers (Optizen POP; Mecasys, Daejeon, Korea and Spectra Max 190 microplate reader; Molecular Devices, CA, USA). Methane was measured by gas chromatography (GC). The detailed condition was described in Section 2.3. Also, the quantity of 16S rRNA and pmoA gene was measured by Q-PCR (CFX96 Real-time System, Bio-rad, Hercules, CA, USA) with primers Bac518F/Bac786R [46] and A189F/A682R [47], respectively. 

### 2.3. Hydrogen Utilization Test

Hydrogen utilization was evaluated in a modified NMS medium with a 2% H_2_ amended or H_2_ nonamended headspace and various methane and oxygen concentration based on Hakobyan et al. [48]. The conditions were as follows. Set1: 20% methane and 15% oxygen; Set2: 20% methane and 3% oxygen; Set3: 6% methane and 15% oxygen; set4: 6% methane and 3% oxygen; and set5: 20% methane and 9% oxygen. Before gas injection, headspace was replaced with Ar gas. Then, all gases were injected with a syringe through a syringe filter (0.22 μm pore size) for sterilization. The hydrogen and methane concentration and OD_600_ were measured during the incubation time.

Methane and hydrogen concentrations in the headspace were monitored by a GC-flame ionization detector/thermal conductivity detector (GC-FID/TCD, Agilent 6890N, Santa Clara, CA, USA) using a 12390-U column. The temperature of the injector was at 200 °C isothermally. The oven temperature was maintained at 65 °C for 1 min, then ramped at a rate of 25 °C/min to 225 °C and held for 2 min. The temperatures of FID and TCD were 250 °C and 230 °C, respectively. The methane and hydrogen were determined with 200 μL of gaseous sample of the headspace of the bottle.

### 2.4. Phylogenetic Analysis

The genome was extracted using a DNA extraction kit (QIAGEN, Valencia, CA, USA). The 16S ribosomal RNA gene was amplified using 27F/1492R primers [49,50], and the methane monooxygenase gene was amplified using A189F/A682R [47], mmox1/mmox2 [51], and mmox206F/mmox886R [52] primers. PCR conditions were as previously described [47,51,52]. Phylogenetic trees based on 16S rRNA genes and *pmoA* genes aligned using Bioedit were constructed using the neighbor-joining method [53] in MEGA 7 [54] based on the Kimura 2-parameter model and Poisson model using 1000 replicates, respectively. The 16S rRNA gene similarity was calculated using EzBioCloud [55].

### 2.5. Genomic Analysis

A paired-end DNA library was constructed using a Truseq Nano DNA kit (Illumina, San Diego, CA, USA). Sequencing was performed by an Illumina HiseqX device at Macrogen (Seoul, Korea). The quality of the raw reads was checked by FastQC. SPAdes (ver. 3.13) was used to assemble the raw reads [56]. The assembled methanotroph scaffolds were annotated using the NCBI Prokaryotic Genome Annotation Pipeline [57]. Gene function was analyzed using BLASTp [58] against the nonredundant (NR) database and the Kyoto Encyclopedia of Genes and Genomes [59]. A domain search was performed using PfamScan [60]. Clusters of orthologous groups (COG) classification was performed according to bac-genomics-scripts (https://github.com/aleimba/bac-genomics-scripts). The average nucleotide identities (ANI) of B8 and other *Methylocystis* species were analyzed using orthologous average nucleotide identity tool (OAT) software [61]. Average amino acid identity (AAI) was calculated using CompareM (https://github.com/dparks1134/CompareM). Pan-genome analysis was performed using PGAP (ver. 1.2.1) [62]. The phylogenomic tree was reconstructed using Anvio-6.1 (default parameter and hmm source: Bacteria_71) [63]. The antibiotic resistance genes were analyzed using a comprehensive antibiotic resistance database (CARD) [64].

### 2.6. Data Availability 

The 16S rRNA gene and draft genome were deposited in the NCBI GenBank as accession numbers MN527243 and VBTZ00000000, respectively.

## 3. Results and Discussion

### 3.1. Information on Sampling Site

The artificial wetland in Ok-Cheon, Republic of Korea (36.19 N, 127.33 E) was constructed to remove nonpoint source pollution from the Daecheong lake, a source of drinking and irrigation water [65]. The water composition of the artificial wetland at the sampling time was summarized in Table 1. The temperature was over 15 °C, except for the winter season [66]. 

### 3.2. Physiological Characterization

Cells of strain B8 were 0.7 μm in width and 1.5 μm in length. Cells were gram-negative, oxidase- and catalase-positive, coccobacilli or short rod-shaped, and appeared as single cells or in aggregates, but not as rosettes. PHB formation was observed by fluorescence microscopy of Nile Red stained sections (Supplementary Figure S1). Colonies did not form on NMS agar, gellan, or phytagel plates. The harvested cell pellet was creamy white. Strain B8 grew between 15 °C and 37 °C, with optimal growth at 30–35 °C. The bacteria were not tolerant to high salinity and grew between 0% to 0.5% NaCl concentration. Growth occurred at pH values ranging from pH 6.5 to 10. The optimal pH was 8.5 to 9, indicating that strain B8 is slightly alkaliphilic. Nitrate ammonia, yeast extract, and cysteine were used as nitrogen sources, while carsitone, casamino acid, and betaine were not. Growth rate was 0.11 ± 0.01 h^−1^ in the modified NMS medium with methane (20%, *v/v*) at 30 °C and 150 rpm. Strain B8 could use methanol at levels ranging from 0.05% to 1%. The optimum level was 0.1%. Ethanol, glucose, and pyruvate were not used as carbon sources. Methane and methanol were the carbon and energy sources for strain B8. In addition, growth was observed using acetate as a carbon and energy source after 1 month of incubation. This result was also reported from other facultative methanotrophs, such as *M. bryophila* H2s, *M. heyeri* H2, *M. echinoides* IMET10491 [39], and *Methylocystis* sp. SB2 [67]. It might serve as a survival strategy in environments with limited methane concentration, such as peat [39]. Table 2 summarizes the findings and compares them with close relatives identified using the phylogenetic analysis described. The physiological properties of strain B8 indicate that this strain is slightly alkaliphilic compared to other members of *Methylocystis.* This might be advantageous in the development of alkaline conditions during photosynthesis in wetlands [68]. Strain B8 was deposited in the Korean Collection for Type Cultures (KCTC) as KCTC82145. 

### 3.3. Hydrogen Utilization and Production

The hydrogen utilization test was conducted using various methane and oxygen conditions. Recently, it was reported that *Methylocystis* sp. SC2 uses hydrogen as an energy source [48]. The growth yield increased significantly under 6% CH_4_ and 3% O_2_ conditions when 2% H_2_ was added to headspace culture. However, the effect was not observed using strain B8. Instead, hydrogen was produced when oxygen decreased to an oxygen limitation level without gas exchange (Supplementary Figure S2). A similar result was found for *Methylomonas* sp. DH-1 [71] and *Methylomicrobium alcaliphilum* 20Z [27], which are type I methanotrophs. GC results showed that this result was related to the type of hydrogenase in *Methylocystis* sp. B8. For additional information, see Section 3.9.

### 3.4. Genomic Features 

The genomic information of strain B8 is shown in Table 3. The G + C content was 59.9% and the lowest in the genomes of the *Methylocystis* species. Genes of the genome were classified according to function using COG analysis (Supplementary Table S1). Genes with ‘no functional prediction’ (S) were most prevalent (13%), followed by those with ‘general function prediction only’ (R, 11%) and related to ‘energy production and conversion’ (C, 8%). Contigs 10 (116,387 bp) and 12 (92,240 bp) displayed a cluster of plasmid replication genes (*repA*, *repB,* and *repC*), respectively. Genes in the clusters FEV16_14560-FEV16_14570 and FEV16_15250-FEV16_15260 of strain B8 were homologous with P1_12-P1_14 and P2_74-76 of *Methylocystis* sp. SC2. These findings indicate that the two contigs belong to a plasmid, further indicating that strain B8 might harbor two large plasmids. Contig 10 also contained mercury reductase genes similar to those of strain SC2 [72]. 

### 3.5. Phylogeny

**Figure 1:**
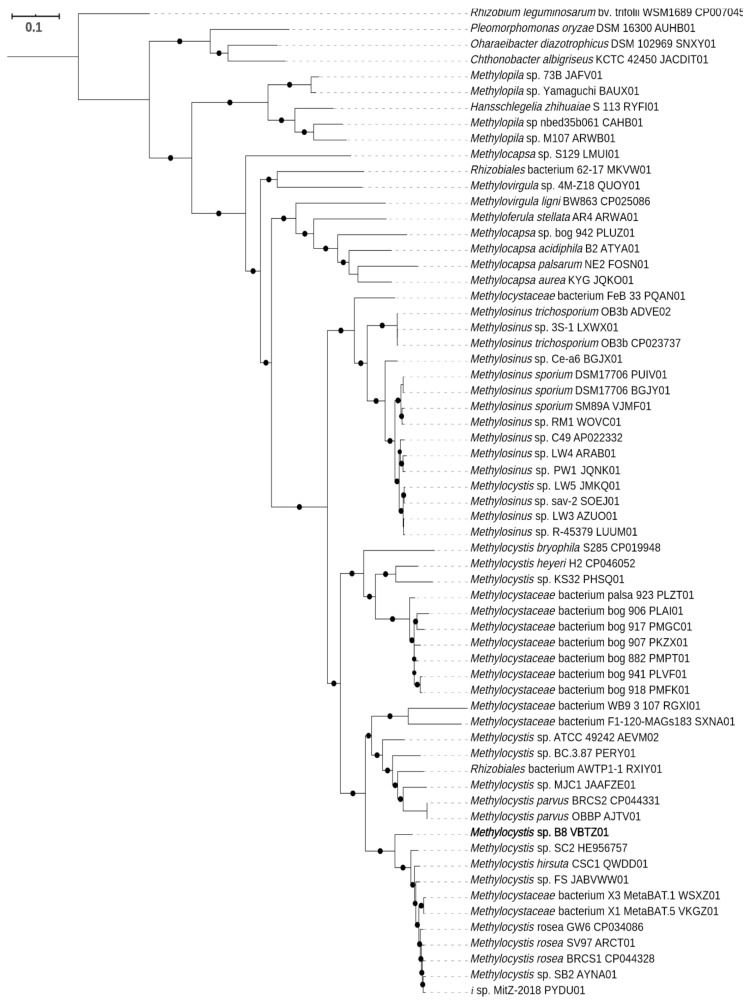
The phylogenetic tree of *Methylocystis* sp. B8 and other strains in the family *Methylocystaceae* based on concatenated conserved proteins. The outgroup was *Rhizobium leguminosarum* bv. *trifolii* in the family *Rhizobiaceae*. Closed circles indicate the bootstrap values ≥70%.

Phylogenetic analysis of the 16S rRNA gene sequence showed that strain B8 clustered with the genus *Methylocystis* in *Alphaproteobacteria*. Strain B8 was closely related to *M. hirsuta* CSC1^T^, *M. rosea* SV97^T^, and *M. echinoides* IMET10491^T^ with 98.8%, 98.4%, and 97.9% 16S rRNA gene similarity, respectively (Supplementary Figure S3). The genome closest to that of strain B8 was *Methylocystis* sp. SC2 (HE956757), with the highest ANI value of 84.76%. The lowest ANI value (73.06) was observed in the genome of *M. bryophila*. The highest and lowest AAI values were obtained with *Methylocystis rosea* BRCS1 (86.9%) and *M*. *bryophila* S285 (68.5%), respectively (Supplementary Table S2). The ANI and AAI results, combined with the phylogenetic analysis results based on 16S rRNA gene sequences, indicate that strain B8 is a novel species in the genus *Methylocystis* based on the threshold values suggested by Konstantidis and Tiedje [73] and Konstantidis et al. [74]. The AAI result showed that genomes of 16 species in *Methylocystis* have been reported or deposited (in NCBI) so far. Furthermore, the phylogenomic tree was reconstructed with genomes of *Methylocystis*, *Methylosinus,* and other genera in the *Methylocystaceae* family (Figure 1). It supported that strain B8 is a new species in the genus *Methylocystis*. Furthermore, we suggest that *Methylocystis* sp. LW5 should be reclassified as a member of the genus *Methylosinus* based on the phylogenomic tree. 

### 3.6. Pangenome Analysis

Eleven strains of *Methylocystis* (Table 3) were compared based on the pan-genome analysis. The *Methylocystis* pan-genome consisted of 13,554 clusters with 1537 core gene clusters in our study. A total of 328 clusters (335 genes) were identified as unique genes in strain B8 (Supplementary Table S3). The COG classification of the unique genes showed that major COGs were assigned to R (general functional prediction), S (function unknown), and M (cell wall, membrane, and envelope biogenesis). As COG M predominantly includes genes for lipopolysaccharide production, these genes might be related to niche adaptation by modifying the surface structures of cells [75]. Recently, another study intensively analyzed the pangenome analysis in the genus *Methylocystis*/*Methylosinus* [76]. Briefly, the genera *Methylocystis* and *Methylosinus* had encoding genes related to methane oxidation and nitrogen fixation. However, their types were different between species. One of the major differences between *Methylocystis* and *Methylosinus* was motility. Furthermore, some species in the genus *Methylocystis* had metabolic flexibility, such as growth on acetate and ethanol as carbon and energy sources, as well as having photosynthetic-related genes.

### 3.7. Methanotrophic Pathway 

**Figure 2:**
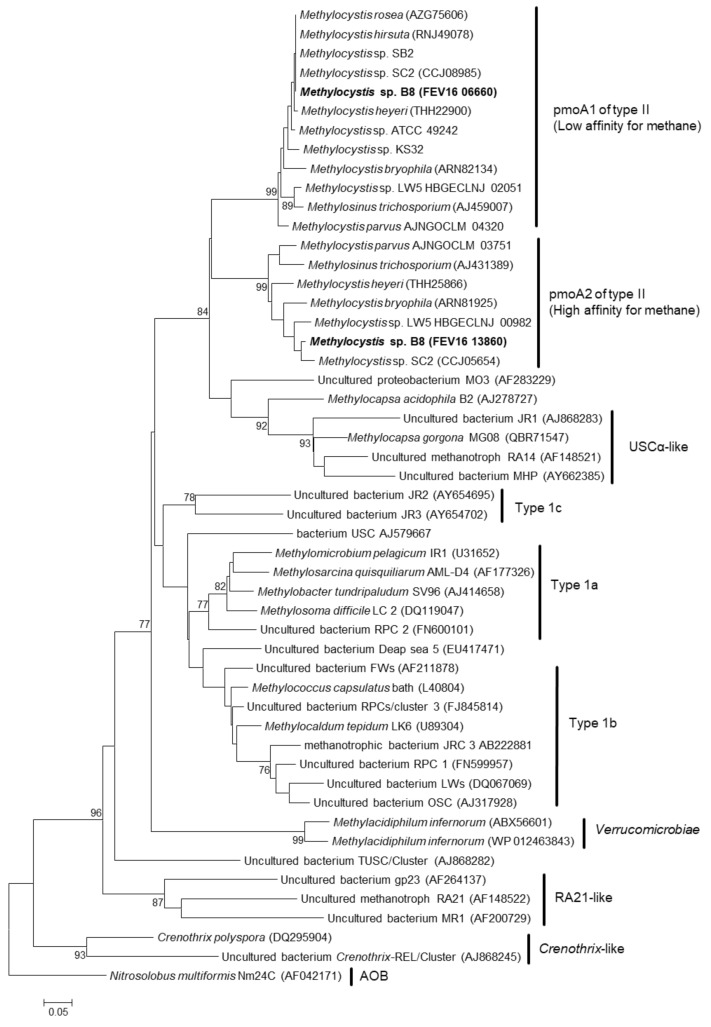
Phylogenetic tree of PmoA of strain B8 and related strains. The phylogenetic tree was reconstructed using the neighbor-joining method. The outgroup was AmoA of *Nitrosolobus multiformis* Mm24C. The clades (right side of the tree) of PmoA was classified according to Cai et al. [79]. Bootstrap values below 70% are not shown. Bar: 0.05 substitutions per amino acid site.

Two gene clusters coding isozymes of pMMO were found in the genome of strain B8. Genes coding for sMMO were not found. The pMMO types were classified using a phylogenic tree (Figure 2). The two pMMO operons, pmoCAB1 and pmoCAB2, coded low-affinity methane oxidation (LAMO) and high-affinity methane oxidation (HAMO), respectively. Additionally, three singleton *pmoC* paralogs were found in the genome of strain B8. Genes related to C_1_ compound metabolism are shown in Supplementary Table S4. Previous studies have reported that additional gene copies of *pmoC* are essential for methanotrophic growth [72,77]. Furthermore, Matsen et al. [78] suggested that the role of PmoC paralogs might be related to the regulation of gene expression or methane sensing, rather than catalytic activity.

The expressions of the HAMO and LAMO genes are regulated by environmental methane concentration [80]. Methanotrophs harboring both HAMO and LAMO enzymes can modify their behavior depending on methane concentration and adapt to various situations. Both types of pMMO were detected in *Methylocystis* sp. SC2, *M. heyeri*, *M. parvus*, and *M. bryophila* S285 (Supplementary Table S5). *M. bryophila*, *M. heyeri*, and *M. hirsuta* also had sMMO. The presence of HAMO and LAMO in the genome of strain B8 might indicate the potential of the strain to survive various methane conditions. In addition, several studies have reported that methanotrophs (especially the *Methylocystis*/*Methylosinus* group) with HAMO activity contribute to the oxidation of atmospheric methane concentrations in soil [79,81]. The utilization of atmospheric methane by strain B8 needs to be demonstrated in a future study.

Methanol dehydrogenase types can be classified depending on whether calcium (*Mxa*) or lanthanides (*Xox*) are required for activity [82,83,84,85]. Both *Mxa,* and *Xox* were detected in strain B8 (Supplementary Table S5). All the evaluated strains, except for KS32, had both *Mxa* and *Xox* (Supplementary Table S5). XoxF reportedly oxidizes methanol to formate rather than formaldehyde [85], although disparate results were recently published [86]. One study reported that the gene expression of Mxa and Xox in *Methylobacter tundripaludum* may change depending on the partner microbes [87]. The function of XoxF in *Methylocystis* requires further study. The XoxF and MxaFI methanol dehydrogenases require pyrroloquinoline quinone (PQQ) as a catalytic cofactor [88,89]. The genome of strain B8 contained the genes *pqqBCE* and *pqqA*, which are required for PQQ biosynthesis. The following steps are described in the Supplementary Information.

### 3.8. Nitrogen Metabolism

Most methanotrophs possess genes related to nitrogen fixation [90,91,92]. Molybdenum-iron (Mo-Fe) nitrogenase was observed in all the genomes of *Methylocystis*. Vanadium-iron (V-Fe) nitrogenases were only found in other methanotrophic bacteria from wetland ecosystems (i.e., *M. heyeri* H2^T^, *M. bryophila* S285, and *M. parvus* BRSC2) [76]. Although strain B8 was obtained from wetlands, it only had genes related to molybdenum-iron (Mo-Fe) nitrogenase. These organisms can assimilate various nitrogen sources, including nitrate, nitrite, and ammonia [34,44,93]. In this study, genes related to nitrogen utilization were found in the genome of strain B8 (Supplementary Table S6). These included assimilatory nitrate/nitrite reduction-related genes, nitrate reductase (FEV16_06420) and nitrite reductase (FEV16_12950) genes, and genes for ammonium transport. These findings suggest that strain B8 could utilize N_2_, nitrate, nitrite, and ammonia as nitrogen sources. Strain B8 also harbored genes coding for hydroxylamine oxidoreductase (FEV16_03625 and FEV16_12730), which oxidizes hydroxylamine to nitrite, and hydroxylamine reductase (FEV16_04040), which catalyzes the reduction of hydroxylamine to ammonia, as observed in *Methylocystis* sp. SC2 [72]. These enzymes have roles in hydroxylamine detoxification as well as nitrogen acquisition.

### 3.9. Hydrogenase

**Figure 3:**
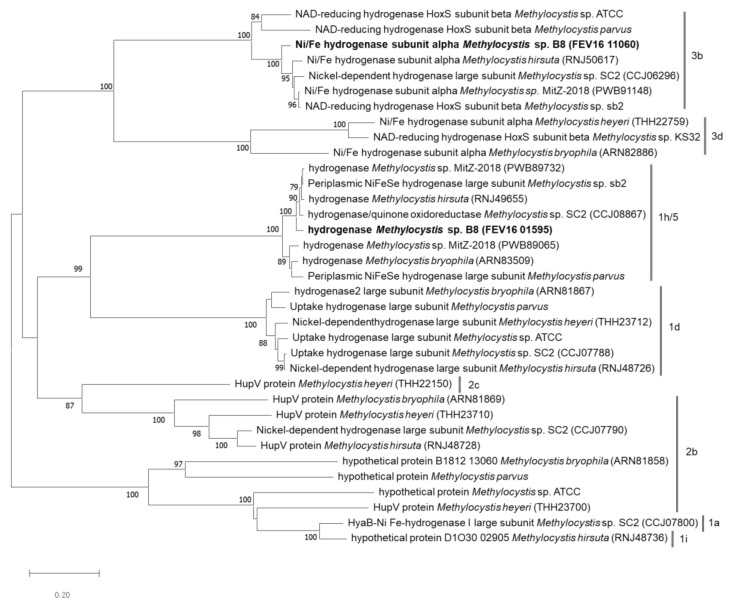
Phylogenetic tree of hydrogenase based on the amino acid sequences of *Methylocystis* sp. B8 and other strains. The tree was constructed with MEGA7 using the neighbor-joining method. These enzymes were classified into eight groups (3b, 3d, 1h/5, 1d, 2c, 2b, 1a, and 1i). Bootstrap values below 70% are not shown. Bar: 0.2 substitutions per amino acid site.

Genes coding for hydrogenase and accessory proteins were observed in all *Methylocystis* genomes except *Methylocystis* sp. KS32. The phylogenetic tree, constructed using the hydrogenase gene sequences (Figure 3), revealed a variation in hydrogenase types within this genus. *Methylocystis* sp. SC2, which is known to use hydrogen as an energy source, had five types of hydrogenase: Ia, Id, 2b, 3b, and 1h/5 group hydrogenase. Among these genes, 1d and 2b group hydrogenases were related to hydrogen utilization by a proteomic study [48]. However, strain B8 had only two groups of hydrogenases, FEV16_01595 and FEV16_11060, which belonged to the 1h/5 and 3b groups, respectively. Nickel (Ni) and iron (Fe) ions are present in both active sites of hydrogenases [94,95,96]. Hydrogen utilization was not observed in strain B8 in our experiments, but hydrogen was produced. The production is hydrogen may have been related to the 3b group hydrogenase. The 3b group hydrogenases reversibly reduce NAD^+^ coupled to H_2_ oxidation [97]. The membrane-bound 1h/5 group hydrogenases are used for aerobic respiration under starvation conditions [97]. It has been hypothesized that hydrogen might be used under starvation conditions. In this study, we demonstrated the hydrogen production potential of B8, a type II methanotroph. The utilization and production of H_2_ in strain B8 need further research in combination with omics studies. The produced hydrogen by methanotrophs in oxygen-limited environments might be an energy source for non-methanotrophs [27]. 

### 3.10. Polyhydroxybutyrate (PHB) Synthesis

Some methanotrophs can synthesize biopolymers, such as PHB, to store carbon and as energy sources [98,99]. Strain B8 was expected to be able to produce and accumulate PHBs because of the presence of genes related to PHB synthesis and degradation. These genes included two poly β-hydroxybutyrate polymerases (FEV16_00425 and 06360), two poly β-hydroxybutyrate depolymerases (FEV16_05615 and 5645), acetyl-CoA acetyltransferase (FEV16_02735), acetoacetyl-CoA reductases (FEV16_02730), polyhydroxyalkanoate synthesis repressors (FEV16_02740), and three phasins (FEV16_07845, 13905, and 15530) (Supplementary Table S7). Storing PHB could lead to a reducing power when cells are starved [99,100]. Biodegradable and biocompatible artificial biopolymers have been developed to replace plastics derived from petroleum [99,101]. The production of PHB-based bioplastics from methane could be advantageous in terms of preventing environmental pollution.

### 3.11. Resistance Genes

Genes for glutaredoxin-dependent arsenate reductase (FEV16_04625, FEV16_04855, and FEV16_04965) and the arsenite efflux pump (FEV16_04970) involved in neutralization or exporting arsenic were demonstrated in strain B8. Arsenic is an especially toxic heavy metal because of its characteristic resemblance to phosphorus [102,103]. Further, arsenic is a redox-sensitive element with a geogenic origin [104]. The detoxification mechanism of arsenic might be necessary for survival in wetlands that feature large redox fluctuations. 

Genes for cyanide detoxification were detected in strain B8. Cyanide is lethal to cells because it interrupts the electron transport systems and halts cellular energy production. Many organisms have defense mechanisms to neutralize cyanide [105,106]. The genes in strain B8 include those coding 3-mercaptopyruvate sulfurtransferase (FEV16_02590 and FEV16_16540) and rhodanese (FEV16_09915, FEV16_10965, and FEV16_13300), which catalyze the conversion of cyanide to thiocyanate. 

Mercury is a toxic heavy metal, and many organisms have a mechanism to neutralize mercury toxicity [107]. Genes related to mercury detoxification, including mercury reductase (FEV16_05105, FEV16_14585, FEV16_14590, FEV16_14595, FEV16_14600, FEV16_15400, and FEV16_15405), were detected in the genome of strain B8. Mercury (II) reductase catalyzes the reduction of mercury (Hg^2+^) to the less toxic elemental mercury (Hg^0^). In addition, methanobactin (*Mbn*) was included in this genome (See Supplementary Information and Supplementary Figure S4). *Mbn* is needed under copper-limited conditions and can also be used for mercury detoxification [108]. In the genus *Methylocystis*, genes for mercury reduction have also been demonstrated in *M*. *rosea*, *Methylocystis* sp. KS32, *M*. *hirsuta*, *Methylocystis* sp. ATCC 49,242, and *Methylocystis* sp. MitZ-2018 (Table 4). The potential for mercury reduction as a means of detoxification in *Methylocystis* sp. HL18 was suggested based on metagenomic analysis [109]. *Methylococcus capsulatus* Bath (type X methanotroph) showed mercury reduction activity [110]. *Methylosinus trichosporium* OB3b can demethylate methylmercury but does not reduce mercury [111]. Further studies are needed to confirm the mercury reduction activity (including the demethylation of methylmercury) in *Methylocystis*.

The genome had genes related to tetracycline and quinolone antibiotic resistance gene (FEV16_04740) based on CARD.

## 4. Conclusions

The physiological and metabolic properties and genomic information of a facultative methanotrophic bacterial strain isolated from a wetland were determined. Phylogenetic/phylogenomic analysis and ANI and AAI values indicated that strain B8 is a novel species in the genus *Methylocystis*. The physiological and genomic analysis demonstrated the versatility of strain B8 in the adaptation to diverse environments. The presence of genes coding HAMO and LAMO indicated that B8 could survive under fluctuating methane concentrations. Strain B8 also harbors genes coding for hydrogenase to produce hydrogen under oxygen-limited conditions. The presence of genes for heavy metal resistance and PHB formation might be useful for survival under stress conditions. These collective features suggest the ubiquitous distribution of strain B8-like methanotrophs and their active roles in methane cycles in wetlands. 

## Figures and Tables

**Table 1:** The properties of sampling water.

Properties	Sampling Water
Sampling date	29 Jun 2017
Temperature (°C)	26.1
pH	6.46
EC (μS/cm)	670
DO (mg/L)	6.82
ORP (mV)	84.7
Ca^2+^ (mg/L)	23.58
K^+^ (mg/L)	16.43
Mg^2+^ (mg/L)	6.49
Na^+^ (mg/L)	83.71
Fluoride (mg/L)	3.19
Chloride (mg/L)	151.56
Nitrite (mg/L)	1.7
Nitrate (mg/L)	26.48
Sulfate (mg/L)	33.22

**Table 2:** Characteristics of *Methylocystis* sp. B8 and related strains in *Methylocystis.*

	1	2	3	4	5	6	7
Cell size (µm)	0.7 × 1.5	0.3–0.6 × 0.7–1.0	0.8–1.1 × 1.1–2.5	0.5 × 0.5–2.0	0.5 × 0.5–1.0	0.9–1.4 × 1.8–3.4	0.8–1.2 × 1.4–4.0
Cell shape	Rod	Dumbbell	Rod	Coccobacillus, rod	Coccobacillus, rod	Small, curved coccoids, short	straight or regularly curved rod, ovoid
Colony color	Cream	Cream	Pink-red	White, buff	Diffusion brown, pale pink	Light cream	White
Temperature (°C)	15–37	<37	5–37	20–28	~20–37	8–37	5–30
Optimum Temperature (°C)	30–35	30	27	25–28	30–37	25–30	25
pH	6.5–10.0	4.0–9.0	5.5–9.0	5.0–9	5.5–8.8	4.2–7.6	4.4–7.5
Optimum pH	8.5–9.0	7.0	NR	6.5–7	7.5	6.0–6.5	5.8–6.2
NaCl (%)	0–0.5	NR	0=<	0–1.0	0–2.0	0–0.1	0–0.5
Optimum NaCl (%)	0–0.5	NR	NR	NR	NR	NR	NR
Oxidase	+	+	+	+	+	+	+
Catalase	+	+	+	+	+	+	+
Methanol (%)	0.05–1	+	-	0.2	5	≤0.15	≤1
Optimum Methanol (%)	0.1	NR	-	NR	NR	NR	0.1
**Carbon Source**							
Ethanol	-	NR	-	NR	NR	±	NR
Acetate	+	w *	-	w *	-	+	w *
Pyruvate	-	NR	NR	NR	NR	±	NR
**Nitrogen Source**							
Cysteine	+	NR	NR	-	-	+	-
Yeast extract	+	NR	NR	+	+	+	+
Casamino acid	-	NR	NR	+	+	NR	NR
Betaine	-	NR	NR	-	-	NR	NR
Carsitone	-	NR	NR	NR	NR	NR	NR
(NH_4_)_2_SO_4_	+	NR	+	+	+	+	+
Nitrate	+	+	+	+	+	+	+

Strains: 1, *Methylocystis* sp. B8 (this study); 2, *Methylocystis hirsuta* CSC1^T^ [31]; 3, *Methylocystis rosea* SV97^T^ [30]; 4, *Methylocystis echinoides* IMET 10491^T^ [28,69]; 5, *Methylocystis parvus* OBBP^T^ [28,70]; 6, *Methylocystis bryophila* H2s^T^ [32,39]; 7, *Methylocystis heyeri* H2^T^ [33]. All strains were gram-negative and non-motile. The characteristics of B8 and other species, *Methylocystis*, are provided. +, Positive reaction; -, negative reaction; w, weakly positive; ±, trace growth; NR, not reported. * Data from Belova et al. [39].

**Table 3:** Genomic features of *Methylocystis* sp. B8 and other strains of *Methylocystis.*

	1	2	3	4	5	6	7	8	9	10	11
Genome size (bp)	3,409,164	4,726,034	4,213,043	3,773,444	3,912,050	4,475,912	3,359,940	4,707,971	4,690,566	4,364,363	3,644,633
GC content	59.9	62.8	62.4	63.4	62.5	63.4	63.1	63.1	63	62.5	62.7
N50 (bp)	386,567	3,131,807	3,776,027	3,773,444	1,614,040	95,607	917.805	4,532,950	3,287,239	136,571	54,643
Coverage (×)	132.6	30	252.0	53	unknown	33	280.0	34.7	53.0	60	2.0
Contig number	28	7	4	1	4	108	9	2	12	55	158
Protein coding genes	3176	4307	4036	3583	3709	4160	3576	4148	3987	4043	3433
rRNA operons	3	6	3	3	3	3	NR	6	5	3	16
tRNA genes	47	53	49	47	50	46	NR	47	54	50	45
Genbankaccession number	VBTZ00000000	AEVM00000000	QWDD00000000	HE956757	ARCT00000000	AJTV00000000	PHSQ00000000	CP019948	SOPH00000000	PYDU00000000	AYNA00000000

Strains: 1, *Methylocystis* sp. B8; 2, *Methylocystis* sp. ATCC 49242; 3, *M. hirsuta* CSC1^T^; 4, *Methylocystis* sp. SC2; 5, *M. rosea* SV97^T^; 6, *M. parvus* OBBP^T^; 7, *Methylocystis* sp. KS32; 8, *M. bryophila* H2s^T^; 9, *M. heyeri* H2^T^; 10, *Methylocystis* sp. MitZ-2018; 11, *Methylocystis* sp. SB2.

**Table 4:** Genes related to heavy metal detoxification in *Methylocystis* sp. B8 and related species.

Heavy Metal Related Genes	1	2	3	4	5	6	7	8	9	10	11	12
Mercury reductase (*merA*)	+	+	+	+	+	-	+	-	-	-	+	-
Arsenate reductase (*arsC*)	+	+	+	+	+	+	+	+	+	+	+	+
Arsenite oxidase	-	-	-	+	-	-	-	-	-	-	-	-
Arsenite methyltransferase	-	-	-	-	-	-	+	-	-	-	-	-
Arsenite efflux transporter	+	-	-	-	+	-	-	-	-	-	-	-
Cobalt-zinc-cadmium resistance protein	-	+	-	-	-	+	+	-	-	+	-	+
Tellurite resistance protein	+	-	+	-	+	-	-	-	+	-	-	-
Copper resistance protein	+	+	+	+	+	+	+	+	+	-	+	+

Strains: 1, *Methylocystis* sp. B8; 2, *Methylocystis* sp. ATCC 49242; 3, *M. hirsuta* CSC1^T^; 4, *Methylocystis* sp. SC2; 5, *M. rosea* SV97^T^; 6, *M. parvus* OBBP^T^; 7, *Methylocystis* sp. KS32; 8, *M. bryophila* H2s^T^; 9, *M. heyeri* H2^T^; 10, *Methylocystis* sp. LW5; 11, *Methylocystis* sp. MitZ-2018; 12, *Methylocystis* sp. SB2. +, positive; -, negative.

## Supplementary Materials

The following are available online at https://www.mdpi.com/2076-2607/8/11/1719/s1. Figure S1: The cells observed with (a) DIC microscopy and (b) fluorescence microscopy stained by Nile red. Figure S2: Monitoring of methane and hydrogen concentrations in headspace of bottles. Methane concentrations in (a) hydrogen non-amended sets and (b) hydrogen-amended sets, hydrogen concentrations in (c) hydrogen non-amended sets and (b) hydrogen-amended sets, and optical density(OD_600_) of (e) non-amended hydrogen sets and (f) hydrogen-amended sets with 200 μL sample using Spectra Max 190 microplate reader. Figure S3: The phylogenetic tree of *Methylocystis* sp. B8 and other strains in the family Methylocystaceae based on the 16S ribosomal RNA gene. The phylogenetic tree was reconstructed using the neighbor-joining method. The outgroup were *Rhizobium leguminosarum* and *Sphingomonas aestuarii* in the family *Rhizobiaceae* and *Sphingomonadaceae* respectively. Bootstrap values below 70% are not shown. Bar, 0.01 substitutions per nucleotide site. Figure S4: Methanobactin gene cluster of strain B8 and other *Methylocystis* species. Table S1. COG classification of the genome of strain B8. Table S2: AAI results of the genera *Methylocystis* and *Methylosinus* (excel). Table S3: COG classification based on the pan-genome and core-genome of the genus *Methylocystis* and the unique clusters of strain B8. Table S4: Genes related to C_1_ compound metabolism in strain B8. Table S5: Comparative analysis of genes related to methane and methanol utilization in *Methylocystis*. Table S6: Genes related to nitrogen metabolism in strain B8. Table S7: Genes related to PHB formation in strain B8.
