# Supplementary material for: Genomic and Physiological Properties of a Facultative Methane-Oxidizing Bacterial Strain of Methylocystis sp. from a Wetland
Source: Microorganisms. 2020 Nov 2;8(11):1719. doi: 10.3390/microorganisms8111719 (PMC7716213; doi:10.3390/microorganisms8111719)
Supplement: Supplementary file 1 [file microorganisms-08-01719-s001.zip › 1.Supplementary_Information.docx]

**Supplementary Information**

Article

Genomic and physiological properties of a facultative methane-oxidizing bacterial strain of *Methylocystis* sp. from a wetland

**Gi-Yong Jung^1, 2^, Sung-Keun Rhee^2^, Young-Soo Han^3^, and So-Jeong Kim^1*^**

^1^ Geologic Environment Research Division, Korea Institute of Geoscience and Mineral Resources, Daejeon 34132, Republic of Korea

^2^ Department of Microbiology, Chungbuk National University, Cheongju 28644, Republic of Korea

^3^ Department of Environmental Engineering, Chungnam National University, Daejeon 34134, Republic of Korea

***** Correspondence: [sojkim86@kigam.re.kr](mailto:sojkim86@kigam.re.kr); Tel: +82-42-868-3311; Fax: +82-42-868-3414

**Tetrahydromethanopterin pathway and serine pathway**

Genes related to the tetrahydromethanopterin-linked pathway of formaldehyde oxidation were detected in strain B8. Formate formation is reportedly the key branch point for dissimilatory and assimilatory metabolism [1]. Like other type II methanotrophs, strain B8 was shown to possess genes encoding NAD-linked enzymes as a dissimilatory formate oxidation. The entry substrate (formate) was converted to methylene-THF by the THF pathway, with C1 then transferred to glycine. Like other type II methanotrophs [2], strain B8 had genes for the serine cycle in an assimilation pathway. In the serine cycle, formate and CO_2_ are assimilated into several amino and organic acids. All the genes for the serine cycle were present in the genome of strain B8 (Supplementary Table S3). They included formate and THF conversion to 5,10-methylene-THF catalyzed by formate-tetrahydrofolate ligase (FEV16_07535), methenyl tetrahydrofolate cyclohydrolase (FEV16_07555), and methylene tetrahydrofolate dehydrogenase (FEV16_07550); serine synthesis from 5,10-methylene-THF and glycine catalyzed by serine hydroxymethyltransferase (FEV16_03890 and FEV16_04755); and glyoxylate regeneration catalyzed by malyl-CoA lyase (FEV16_07575 and FEV16_09390).

**Methanobactin**

The gene for the production of methanobactin (*Mbn*) was identified in the B8 genome. The group IIa Mbn operon of strain B8 was similar to that of *M. rosea* SV97, *Methylocystis* sp. SC2, *Methylocystis* sp. Miz-2018, and *Methylocystis* sp. SB2 in gene organization (Supplementary Figure S4). These strains had the same precursor peptide sequence (MbnA), with the exception of *Methylocystis* sp. SC2. The Mbn precursor was not observed in the genomes of KS32, *M. heyeri*, or *Methylocystis* sp. ATCC 49242.

**References**

1. Crowther, G.J.; Kosaly, G.; Lidstrom, M.E. Formate as the main branch point for methylotrophic metabolism in *Methylobacterium extorquens* AM1. *J Bacteriol* **2008**, *190*, 5057-5062, doi:10.1128/JB.00228-08.

2. Higgins, I.; Best, D.; Hammond, R.; Scott, D. Methane-oxidizing microorganisms. *Microbiol Rev* **1981**, *45*, 556.
