# Supplementary material for: Genomic and Physiological Properties of a Facultative Methane-Oxidizing Bacterial Strain of Methylocystis sp. from a Wetland
Source: Microorganisms. 2020 Nov 2;8(11):1719. doi: 10.3390/microorganisms8111719 (PMC7716213; doi:10.3390/microorganisms8111719)
Supplement: Supplementary file 1 [file microorganisms-08-01719-s001.zip › 2.Supplementary_Tables.docx]

**Table S1.** COG classification of the genome of strain B8

| **Category letter** | **Function** | **The number of genes** |
| --- | --- | --- |
|  | **CELLULAR PROCESSES AND SIGNALING** |  |
| D | Cell cycle control, cell division, chromosome partitioning | 37 |
| M | Cell wall/membrane/envelope biogenesis | 180 |
| N | Cell motility | 7 |
| O | Post-translational modification, protein turnover and chaperones | 135 |
| T | Signal transduction mechanisms | 119 |
| U | Intracellular trafficking, secretion, and vesicular transport | 54 |
| V | Defense mechanisms | 26 |
| W | Extracellular structures | 0 |
| Y | Nuclear structure | 1 |
| Z | Cytoskeleton | 1 |
|  | **INFORMATION STORAGE AND PROCESSING** | |
| A | RNA processing and modification | 0 |
| B | Chromatin structure and dynamics | 2 |
| J | Translation, ribosomal structure, and biogenesis | 165 |
| K | Transcription | 136 |
| L | Replication, recombination, and repair | 123 |
|  | **METABOLISM** |  |
| C | Energy production and conversion | 207 |
| E | Amino acid transport and metabolism | 190 |
| F | Nucleotide transport and metabolism | 56 |
| G | Carbohydrate transport and metabolism | 98 |
| H | Coenzyme transport and metabolism | 146 |
| I | Lipid transport and metabolism | 120 |
| P | Inorganic ion transport and metabolism | 152 |
| Q | Secondary metabolites biosynthesis, transport, and catabolism | 60 |
|  | **POORLY CHARACTERIZED** |  |
| R | General function prediction only | 294 |
| S | Function unknown | 338 |

**Table S2.** AAI results of the genera *Methylocystis* and *Methylosinus* (excel).

**Table S3.** COG classification based on the pan-genome and core-genome of the genus *Methylocystis* and the unique clusters of strain B8.

| **Function** | **Pan genome** | **Core genome** | **The number of unique clusters** |
| --- | --- | --- | --- |
| **CELLULAR PROCESSES AND SIGNALING** |  |  |  |
| Cell cycle control, cell division, chromosome partitioning | 104 | 17 | 2 |
| Cell wall/membrane/envelope biogenesis | 625 | 86 | 23 |
| Cell motility | 117 | 2 | 1 |
| Post-translational modification, protein turnover  and chaperones | 273 | 81 | 5 |
| Signal transduction mechanisms | 445 | 62 | 4 |
| Intracellular trafficking, secretion and vesicular transport | 213 | 25 | 5 |
| Defense mechanisms | 155 | 14 | 2 |
| Extracellular structures | 3 | 0 | 0 |
| Cytoskeleton | 6 | 0 | 0 |
| **INFORMATION STORAGE AND PROCESSING** |  |  |  |
| Chromatin structure and dynamics | 2 | 1 | 0 |
| Translation, ribosomal structure and biogenesis | 217 | 131 | 0 |
| Transcription | 540 | 57 | 7 |
| Replication, recombination and repair | 572 | 71 | 7 |
| **METABOLISM** |  |  |  |
| Energy production and conversion | 442 | 121 | 5 |
| Amino acid transport and metabolism | 377 | 129 | 6 |
| Nucleotide transport and metabolism | 93 | 49 | 2 |
| Carbohydrate transport and metabolism | 307 | 51 | 5 |
| Coenzyme transport and metabolism | 309 | 104 | 7 |
| Lipid transport and metabolism | 261 | 78 | 5 |
| Inorganic ion transport and metabolism | 432 | 63 | 4 |
| Secondary metabolites biosynthesis, transport and catabolism | 184 | 30 | 3 |
| **POORLY CHARACTERIZED** |  |  |  |
| General function prediction only | 981 | 139 | 26 |
| Function unknown | 1080 | 149 | 22 |
| **Unclassified** | 5813 | 77 | 185 |

**Table S4.** Genes related to C_1_ compound metabolism in strain B8.

| **Locus tag** | **Protein/function** | **Gene name** |
| --- | --- | --- |
| **Methane monooxygenase** | |  |
| FEV16_02225 | Particulate methane monooxygenase subunit C1 | *pmoC2* |
| FEV16_04865 | Particulate methane monooxygenase subunit C1,  partial | *pmoC1* |
| FEV16_06660 | Particulate methane monooxygenase subunit A1 | *pmoA* |
| FEV16_06655 | Particulate methane monooxygenase subunit B1 | *pmoB* |
| FEV16_13465 | Particulate methane monooxygenase subunit C1 | *pmoC* |
| FEV16_13855 | Particulate methane monooxygenase subunit C2 | *pmoC2* |
| FEV16_13860 | Particulate methane monooxygenase subunit A2 | *pmoA2* |
| FEV16_13865 | Particulate methane monooxygenase subunit B2 | *pmoB2* |
| FEV16_16770 | Particulate methane monooxygenase subunit C1,  partial | *pmoC1* |
| **Methanol dehydrogenase** | |  |
| FEV16_08445 | PQQ-dependent dehydrogenase | *mxaF* |
| FEV16_08450 | Methanol oxidation system protein | *moxJ* |
| FEV16_08455 | Cytochrome *c*-L | *mxaG* |
| FEV16_08460 | Methanol dehydrogenase small beta subunit | *mxaI* |
| FEV16_08465 | MoxR family ATPase | *moxR* |
| FEV16_08470 | MxaS protein, involved in methanol oxidation | *mxaS* |
| FEV16_08475 | MxaA protein involved in Ca^2+^ insertion into  methanol dehydrogenase | *mxaA* |
| FEV16_08480 | MxaC protein involved in Ca^2+^ insertion into  methanol dehydrogenase | *mxaC* |
| FEV16_08485 | MxaK protein involved in Ca^2+^ insertion into  methanol dehydrogenase | *mxaK* |
| FEV16_08490 | MxaL protein involved in Ca^2+^ insertion into methanol dehydrogenase | *mxaL* |
| FEV16_08495 | MxaD-like protein | *mxaD* |
| FEV16_08500 | Putative MxaH-like protein | *mxaH* |
| FEV16_09920 | Putative methanol oxidation protein | *xoxJ* |
| FEV16_09925 | C-type cytochrome methanol metabolism-related | *xoxG* |
| FEV16_09930 | PQQ-dependent dehydrogenase | *xoxF* |
| **Pyrroloquinoline quinone (PQQ) biosynthesis** | |  |
| FEV16_05065 | Pyrroloquinoline quinone biosynthesis protein B | *pqqB* |
| FEV16_05070 | Pyrroloquinoline quinone biosynthesis protein C | *pqqC* |
| FEV16_05075 | Pyrroloquinoline quinone biosynthesis protein E | *pqqE* |
| FEV16_05235 | pyrroloquinoline quinone precursor peptide | *pqqA* |
| FEV16_14685 | pyrroloquinoline quinone precursor peptide | *pqqA* |
| **Tetrahydromethanopterin pathway** | |  |
| FEV16_01020 | NAD(P)-dependent methylenetetrahydromethanopterin  dehydrogenase | *mtd* |
| FEV16_01025 | Formaldehyde-activating enzyme | *Fae* |
| FEV16_01030 | H_4_MPT-linked C_1_ transfer pathway protein |  |
| FEV16_02340 | formaldehyde-activating enzyme | *Fae* |
| FEV16_05215 | Formylmethanofuran dehydrogenase subunit C | *fwdC* |
| FEV16_05220 | Formylmethanofuran—tetrahydromethanopterin  N-Formyltransferase | *ftr* |
| FEV16_05225 | Formylmethanofuran dehydrogenase subunit A | *fwdA* |
| FEV16_05230 | Formylmethanofuran dehydrogenase | *fwdB* |
| FEV16_09895 | Formaldehyde-activating enzyme | *Fae* |
| FEV16_09900 | Formaldehyde-activating enzyme | *Fae* |
| FEV16_13475 | Methenyltetrahydromethanopterin cyclohydrolase | *mch* |
| FEV16_13490 | Aldehyde-activating enzyme | *fae* |
| **Serine cycle** | | |
| FEV16_07535 | formate-tetrahydrofolate ligase | *fhs* |
| FEV16_07550 | methylenetetrahydrofolate dehydrogenase | *folD* |
| FEV16_07555 | methenyltetrahydrofolate cyclohydrolase | *folD* |
| FEV16_03890 | serine hydroxymethyltransferase | *glyA* |
| FEV16_04755 | serine hydroxymethyltransferase | *glyA* |
| FEV16_07540 | serine-pyruvate aminotransferase | - |
| FEV16_07545 | glycerate dehydrogenase | *hprA* |
| FEV16_10670 | D-glycerate dehydrogenase | *hprA* |
| FEV16_07580 | glycerate kinase | *gck* |
| FEV16_05740 | phosphopyruvate hydratase | - |
| FEV16_07570 | phosphoenolpyruvate carboxylase | - |
| FEV16_13305 | phosphoenolpyruvate carboxylase | - |
| FEV16_00590 | malate dehydrogenase | - |
| FEV16_07560 | malate-CoA ligase | - |
| FEV16_07575 | malyl-CoA lyase | - |
| FEV16_09390 | malyl-CoA lyase | - |
| **Formate oxidation** | |  |
| FEV16_06455 | Formate hydrogenlyase | *fdwA* |
| FEV16_07920 | Formate dehydrogenase subunit gamma | *fdsG* |
| FEV16_07925 | Formate dehydrogenase subunit beta | *fdwB* |
| FEV16_07930 | NAD-dependent formate dehydrogenase,  alpha subunit | - |
| FEV16_07940 | NAD-dependent formate dehydrogenase  delta subunit | - |
| FEV16_07935 | Formate dehydrogenase family accessory protein | - |
| FEV16_15285 | NAD-dependent formate dehydrogenase | *fdh* |
| **Aromatic hydrocarbon compound degradation** | |  |
| FEV16_13550 | Hydrocarbon degradation protein | *faDL* |

**Table S5.** Comparative analysis of genes related to methane and methanol utilization in *Methylocystis*

Strains: 1, *Methylocystis* sp. B8; 2, *Methylocystis* sp*.* ATCC 49242; 3, *M. hirsuta* CSC1^T^; 4, *Methylocystis* sp*.* SC2; 5, *M. rosea* SV97^T^; 6, *M. parvus* OBBP^T^; 7, *Methylocystis* sp*.* KS32; 8, *M. bryophila* H2s^T^; 9, *M. heyeri* H2^T^; 10, *Methylocystis* sp*.* MitZ-2018; 11, *Methylocystis* sp*.* SB2. +, positive; -, negative.

| **Gene name** | **1** | **2** | **3** | **4** | **5** | **6** | **7** | **8** | **9** | **11** | **12** |
| --- | --- | --- | --- | --- | --- | --- | --- | --- | --- | --- | --- |
| *pmoC1* | + | + | + | + | + | + | + | + | + | + | + |
| *pmoA1* | + | + | + | + | + | + | + | + | + | + | + |
| *pmoB1* | + | + | + | + | + | + | + | + | + | + | + |
| *pmoC2* | + | - | - | + | - | + | - | + | + | - | - |
| *pmoA2* | + | - | - | + | - | + | - | + | + | - | - |
| *pmoB2* | + | - | - | + | - | + | - | + | + | - | - |
| Cu-MMO protein family PxmA | - | - | + | - | + | - | - | + | - | + | + |
| Cu-MMO protein family PxmB | - | - | + | - | + | - | - | + | - | + | + |
| Cu-MMO protein family PxmC | - | - | + | - | + | - | - | + | - | + | + |
| *mxaF* | + | + | + | + | + | + | + | + | + | + | + |
| *mxaJ* | + | + | + | + | + | + | + | + | + | + | + |
| *mxaG* | + | + | + | + | + | + | - | + | + | + | + |
| *mxaI* | + | + | + | + | + | + | - | - | + | + | + |
| *mxaR* | + | + | + | + | + | + | + | + | + | + | + |
| *mxaS* | + | + | + | + | + | + | - | + | + | + | + |
| *mxaA* | + | - | + | + | + | + | - | + | + | + | + |
| *mxaC* | + | + | + | + | + | + | - | + | + | + | + |
| *mxaK* | + | + | + | + | + | + | - | + | + | + | + |
| *mxaL* | + | + | + | + | + | + | - | + | + | + | + |
| *mxaD* | + | + | + | + | + | + | + | + | + | + | + |
| *mxaH* | + | + | + | + | + | - | - | - | - | + | + |
| *xoxJ* | + | + | + | + | + | + | + | + | + | + | + |
| *xoxG* | + | + | + | + | + | + | + | + | + | + | + |
| *xoxF* | + | + | + | + | + | + | + | + | + | + | + |

**Table S6.** Genes related to nitrogen metabolism in strain B8.

| **Locus tag** | **Protein/ function** | **Gene name** |
| --- | --- | --- |
| **Nitrogen fixation** | |  |
| FEV16_16330 | Putative ferredoxin protein | *fixX* |
| FEV16_16335 | Flavoprotein-ubiquinone oxidoreductase | *fixC* |
| FEV16_16340 | Electron transfer flavoprotein, alpha subunit | *fixB* |
| FEV16_16345 | Electron transfer flavoprotein beta-subunit | *fixA* |
| FEV16_16350 | Nitrogenase-stabilizing/protective protein | *nifW* |
| FEV16_16355 | NifP protein, serine O-acetyltransferase | *-* |
| FEV16_16360 | NifV protein, encodes a homocitrate synthase | *nifV* |
| FEV16_16365 | NifU-like iron-sulfur cluster assembly protein | *-* |
| FEV16_16370 | Putative nitrogen fixation protein | *nifQ* |
| FEV16_16375 | Nitrogenase iron protein | *nifH* |
| FEV16_16500 | Nitrogen fixation protein | *fixU* |
| FEV16_16505 | Nitrogenase metalloclusters biosynthesis protein | *nifS* |
| FEV16_16510 | Putative NifU protein | *nifU* |
| FEV16_16560 | NifX-associated protein | *-* |
| FEV16_16565 | NifX-associated protein | *-* |
| FEV16_16570 | Nitrogen fixation protein | *nifX* |
| FEV16_16575 | Nitrogenase molybdenum-iron cofactor biosynthesis protein | *nifN* |
| FEV16_16580 | Nitrogenase MoFe cofactor biosynthesis protein | *nifE* |
| FEV16_16585 | Nitrogenase molybdenum-iron protein beta chain | *nifD* |
| FEV16_16590 | Nitrogenase protein alpha chain | *nifD* |
| FEV16_16595 | Nitrogenase iron protein | *nifH* |
| FEV16_16665 | NifZ family protein | *-* |
| FEV16_16670 | NifZ family protein | *-* |
| FEV16_16675 | LRV FeS4 cluster domain protein | *-* |
| FEV16_16680 | 4Fe-4S ferredoxin, nitrogenase-associated protein | *-* |
| FEV16_16685 | Nif-specific regulatory protein | *nifA* |
| FEV16_16715 | Nitrogenase reductase | *nifH* |
| FEV16_16720 | LRV FeS cluster domain protein | *-* |
| FEV16_16730 | Putative iron-sulfur cluster assembly protein | *-* |
| FEV16_16735 | Ferredoxin-like protein | *-* |
| FEV16_16745 | Nitrogenase FeMo cofactor biosynthesis protein | *nifB* |
| **Nitrate/nitrite assimilation** | |  |
| FEV16_02770 | Nitrate ABC transporter permease | *nrtB* |
| FEV16_02775 | Nitrate ABC transporter ATP-binding protein | *nrtC* |
| FEV16_02780 | Nitrate ABC transporter substrate-binding protein | *nrtA* |
| FEV16_06420 | Nitrate reductase | *-* |
| FEV16_12945 | Nitrate transporter component | *nrtA* |
| FEV16_12950 | Nitrite reductase [NAD(P)H], large subunit | *nasD* |
| FEV16_12955 | NAD(P)H-dependent nitrite reductase catalytic subunit | *nirA* |
| FEV16_12960 | NAD(P)H-dependent nitrite reductase flavoprotein subunit | *-* |
| FEV16_12970 | Nitrate transporter | *narK* |
| **Ammonium transporter** | |  |
| FEV16_12280 | Putative ammonium transporter | *-* |
| FEV16_12335 | Putative ammonium transporter | *-* |
| **Ammonia assimilation** | |  |
| FEV16_02580 | Glutamine synthetase (GS) | *-* |
| FEV16_03655 | Glutamate synthetase (GOGAT), large subunit | *-* |
| FEV16_03665 | Glutamate synthetase (GOGAT), small subunit | *-* |
| **Nitrogen metabolism-related regulatory components** | |  |
| FEV16_08790 | RNA polymerase sigma factor RpoN | *rpoN* |
| FEV16_13960 | tRNA-dihydrouridine synthase | *nifR3* |
| FEV16_13965 | Signal transduction histidine kinase, nitrogen specific | *ntrB* |
| FEV16_13970 | Nitrogen metabolism transcriptional regulator | *ntrC* |
| FEV16_13975 | Multi-sensor signal transduction histidine kinase | *ntrY* |
| FEV16_13980 | Nitrogen assimilation regulatory protein | *ntrX* |
| **Nitrification/ hydroxylamine detoxification** | |  |
| FEV16_03625 | Hydroxylamine oxidoreductase subunit | *haoB* |
| FEV16_04040 | Hydroxylamine reductase | *hcp* |
| FEV16_12730 | Hydroxylamine oxidoreductase subunit | *haoA* |
| **Denitrification** | |  |
| FEV16_04910 | Copper ABC transporter ATP-binding protein | *nosF* |
| FEV16_07400 | NnrU family protein (probable denitrification associated gene) | *-* |

**Table S7.** Genes related to PHB formation in strain B8.

| **Accession number** | **Protein/ function** | **Gene name** |
| --- | --- | --- |
| FEV16_00425 | Class I poly(R)-hydroxyalkanoic acid synthase | *phaC* |
| FEV16_02730 | acetoacetyl-CoA reductase | *phbB* |
| FEV16_02735 | acetoacetyl-CoA reductase | *phbA* |
| FEV16_02740 | Polyhydroxyalkanoate synthesis repressor PhaR | *phaR* |
| FEV16_05615 | polyhydroxyalkanoate depolymerase | - |
| FEV16_05645 | Polyhydroxyalkanoate depolymerase | - |
| FEV16_06360 | Poly(3-hydroxyalkanoate) synthetase | - |
| FEV16_07845 | Phasin | - |
| FEV16_13905 | Phasin family protein | - |
| FEV16_15530 | Phasin family protein | - |

**Supplementary Figure Legend**

**Figure S1.** The cells observed with a) DIC microscopy and b) fluorescence microscopy stained by Nile red.

Figure S2. Monitoring of methane and hydrogen concentrations in headspace of bottles. Methane concentrations in a) hydrogen non-amended sets and b) hydrogen-amended sets, hydrogen concentrations in c) hydrogen non-amended sets and b) hydrogen-amended sets, and optical density(OD_600_) of e) non-amended hydrogen sets and f) hydrogen-amended sets with 200 μl sample using Spectra Max 190 microplate reader.

**Fig. S3.** The phylogenetic tree of *Methylocystis* sp. B8 and other strains in the family *Methylocystaceae* based on the 16S ribosomal RNA gene. The phylogenetic tree was reconstructed using the neighbor-joining method. The outgroup were *Rhizobium leguminosarum* and *Sphingomonas aestuarii* in the family *Rhizobiaceae* and *Sphingomonadaceae*, respectively. Bootstrap values below 70% are not shown. Bar, 0.01 substitutions per nucleotide site.

**Fig. S4.** Methanobactin gene cluster of strain B8 and other *Methylocystis* species.
