# Supplementary material for: Genomic and Physiological Properties of a Facultative Methane-Oxidizing Bacterial Strain of Methylocystis sp. from a Wetland
Source: Microorganisms. 2020 Nov 2;8(11):1719. doi: 10.3390/microorganisms8111719 (PMC7716213; doi:10.3390/microorganisms8111719)
Supplement: Supplementary file 1 [file microorganisms-08-01719-s001.zip › 5.Supplementary_Figure_2.pptx]

## Slide 1
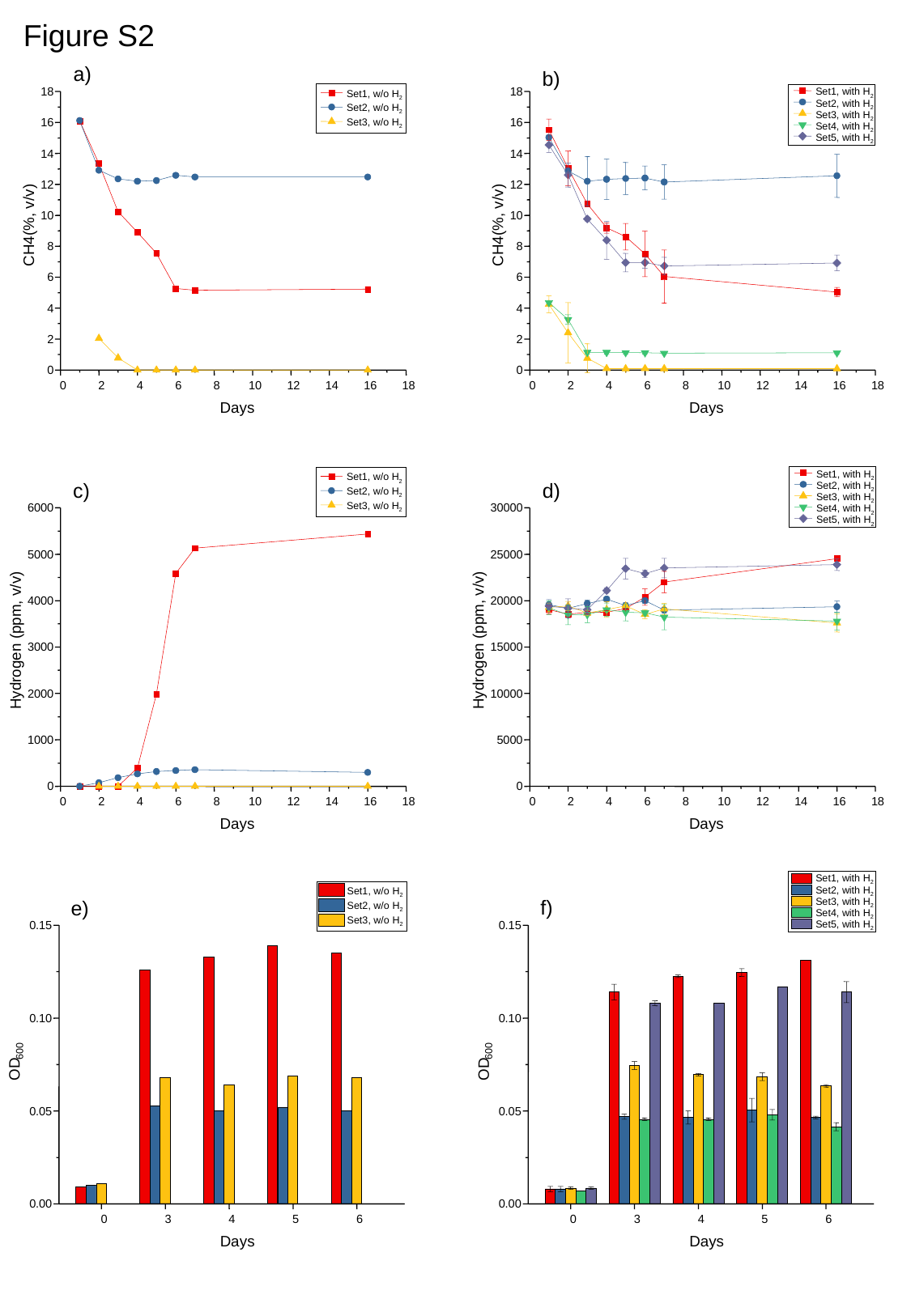

Figure S2
18
Set1, w/o H2
Set2, w/o H2
16
Set3, w/o H2
14
12
10
CH4(%, v/v)
8
6
4
2
0
0
2
4
6
8
10
12
14
16
18
Days
18
16
14
12
10
8
6
4
2
0
0
2
4
6
8
10
12
14
16
18
 Set1, with H2
 Set2, with H2
 Set3, with H2
 Set4, with H2
 Set5, with H2
CH4(%, v/v)
Days
a)
b)
6000
5000
4000
Hydrogen (ppm, v/v)
3000
2000
1000
0
0
2
4
6
8
10
12
14
16
18
Days
30000
25000
20000
15000
10000
5000
0
0
2
4
6
8
10
12
14
16
18
Hydrogen (ppm, v/v)
Days
 Set1, with H2
Set1, w/o H2
d)
c)
 Set2, with H2
Set2, w/o H2
 Set3, with H2
Set3, w/o H2
 Set4, with H2
 Set5, with H2
 Set1, with H2
0.15
0.10
600
OD
0.05
0.00
0
3
4
5
6
Days
0.15
0.10
600
OD
0.05
0.00
0
3
4
5
6
Days
 Set2, with H2
Set1, w/o H2
f)
e)
 Set3, with H2
Set2, w/o H2
 Set4, with H2
Set3, w/o H2
 Set5, with H2
