# Supplementary material for: Genomic and Physiological Properties of a Facultative Methane-Oxidizing Bacterial Strain of Methylocystis sp. from a Wetland
Source: Microorganisms. 2020 Nov 2;8(11):1719. doi: 10.3390/microorganisms8111719 (PMC7716213; doi:10.3390/microorganisms8111719)
Supplement: Supplementary file 1 [file microorganisms-08-01719-s001.zip › 6.Supplementary_Figure_3.pptx]

## Slide 1
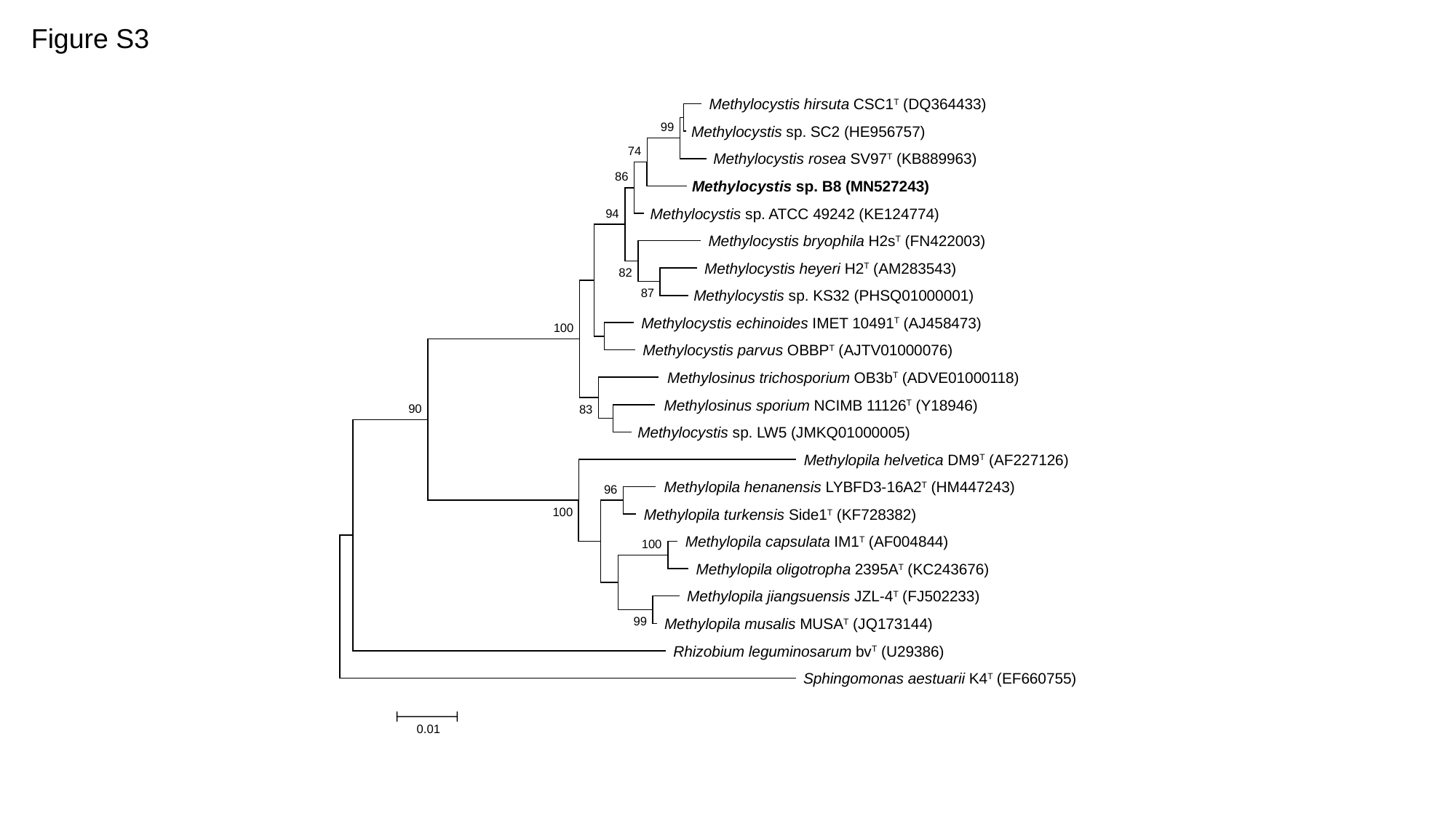

Figure S3
 Methylocystis hirsuta CSC1T (DQ364433)
99
 Methylocystis sp. SC2 (HE956757)
74
 Methylocystis rosea SV97T (KB889963)
86
 Methylocystis sp. B8 (MN527243)
 Methylocystis sp. ATCC 49242 (KE124774)
94
 Methylocystis bryophila H2sT (FN422003)
 Methylocystis heyeri H2T (AM283543)
82
87
 Methylocystis sp. KS32 (PHSQ01000001)
 Methylocystis echinoides IMET 10491T (AJ458473)
100
 Methylocystis parvus OBBPT (AJTV01000076)
 Methylosinus trichosporium OB3bT (ADVE01000118)
 Methylosinus sporium NCIMB 11126T (Y18946)
90
83
 Methylocystis sp. LW5 (JMKQ01000005)
 Methylopila helvetica DM9T (AF227126)
 Methylopila henanensis LYBFD3-16A2T (HM447243)
96
100
 Methylopila turkensis Side1T (KF728382)
 Methylopila capsulata IM1T (AF004844)
100
 Methylopila oligotropha 2395AT (KC243676)
 Methylopila jiangsuensis JZL-4T (FJ502233)
99
 Methylopila musalis MUSAT (JQ173144)
 Rhizobium leguminosarum bvT (U29386)
 Sphingomonas aestuarii K4T (EF660755)
0.01
