# Supplementary material for: Genomic and Physiological Properties of a Facultative Methane-Oxidizing Bacterial Strain of Methylocystis sp. from a Wetland
Source: Microorganisms. 2020 Nov 2;8(11):1719. doi: 10.3390/microorganisms8111719 (PMC7716213; doi:10.3390/microorganisms8111719)
Supplement: Supplementary file 1 [file microorganisms-08-01719-s001.zip › 7.Supplementary_Figure_4.pptx]

## Slide 1
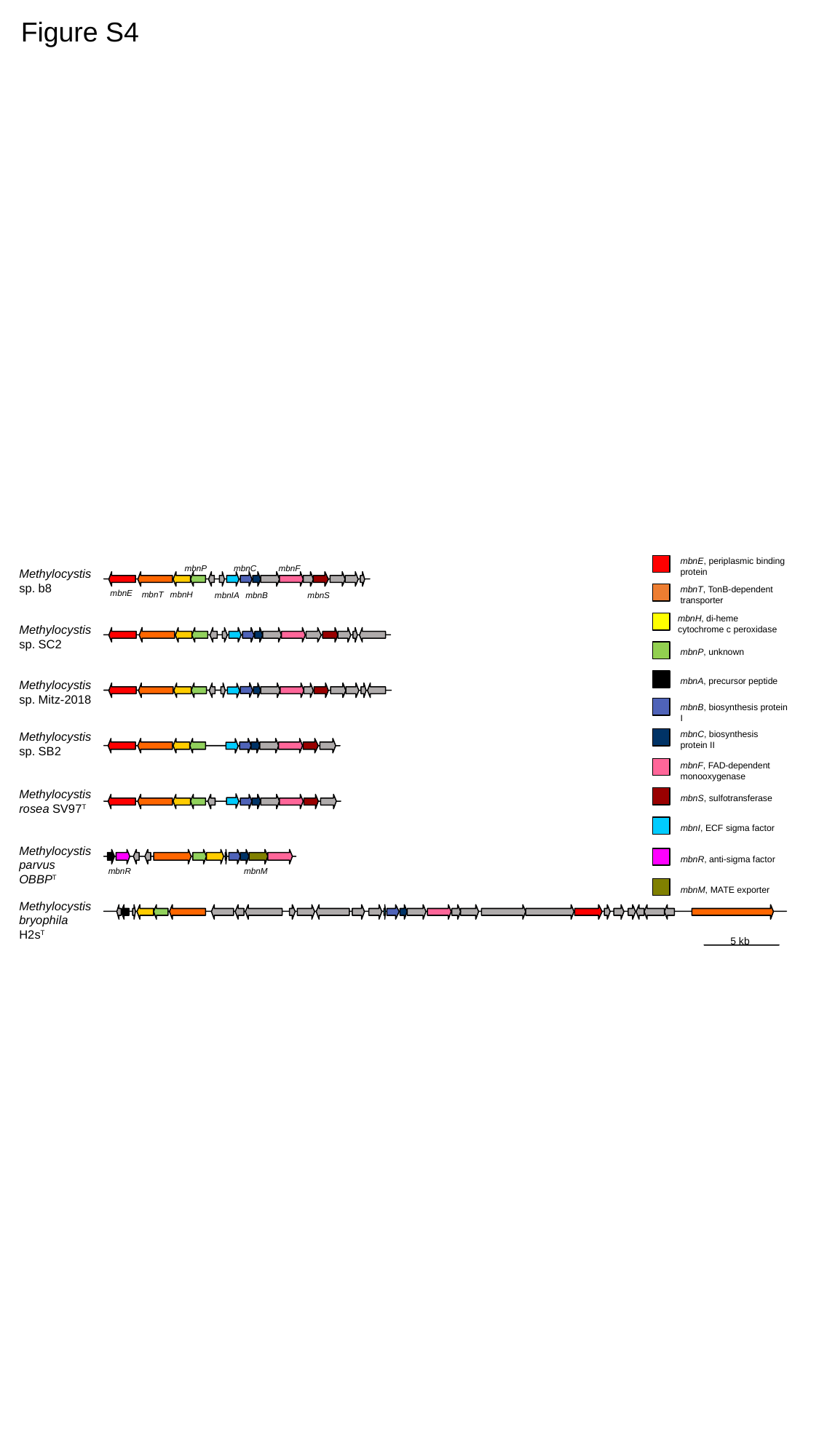

Figure S4
mbnE, periplasmic binding
protein
mbnP
mbnC
mbnF
Methylocystis sp. b8
mbnE
mbnT
mbnH
mbnS
mbnB
mbnIA
mbnT, TonB-dependent
transporter
mbnH, di-heme cytochrome c peroxidase
Methylocystis sp. SC2
mbnP, unknown
mbnA, precursor peptide
Methylocystis sp. Mitz-2018
mbnB, biosynthesis protein I
mbnC, biosynthesis protein II
Methylocystis sp. SB2
mbnF, FAD-dependent monooxygenase
Methylocystis rosea SV97T
mbnS, sulfotransferase
mbnI, ECF sigma factor
Methylocystis parvus OBBPT
mbnR, anti-sigma factor
mbnR
mbnM
mbnM, MATE exporter
Methylocystis bryophila H2sT
5 kb
