# Supplementary figures and images for: Genomic and Physiological Properties of a Facultative Methane-Oxidizing Bacterial Strain of Methylocystis sp. from a Wetland
Source: Microorganisms. 2020 Nov 2;8(11):1719. doi: 10.3390/microorganisms8111719 (PMC7716213; doi:10.3390/microorganisms8111719)

## Slide 1
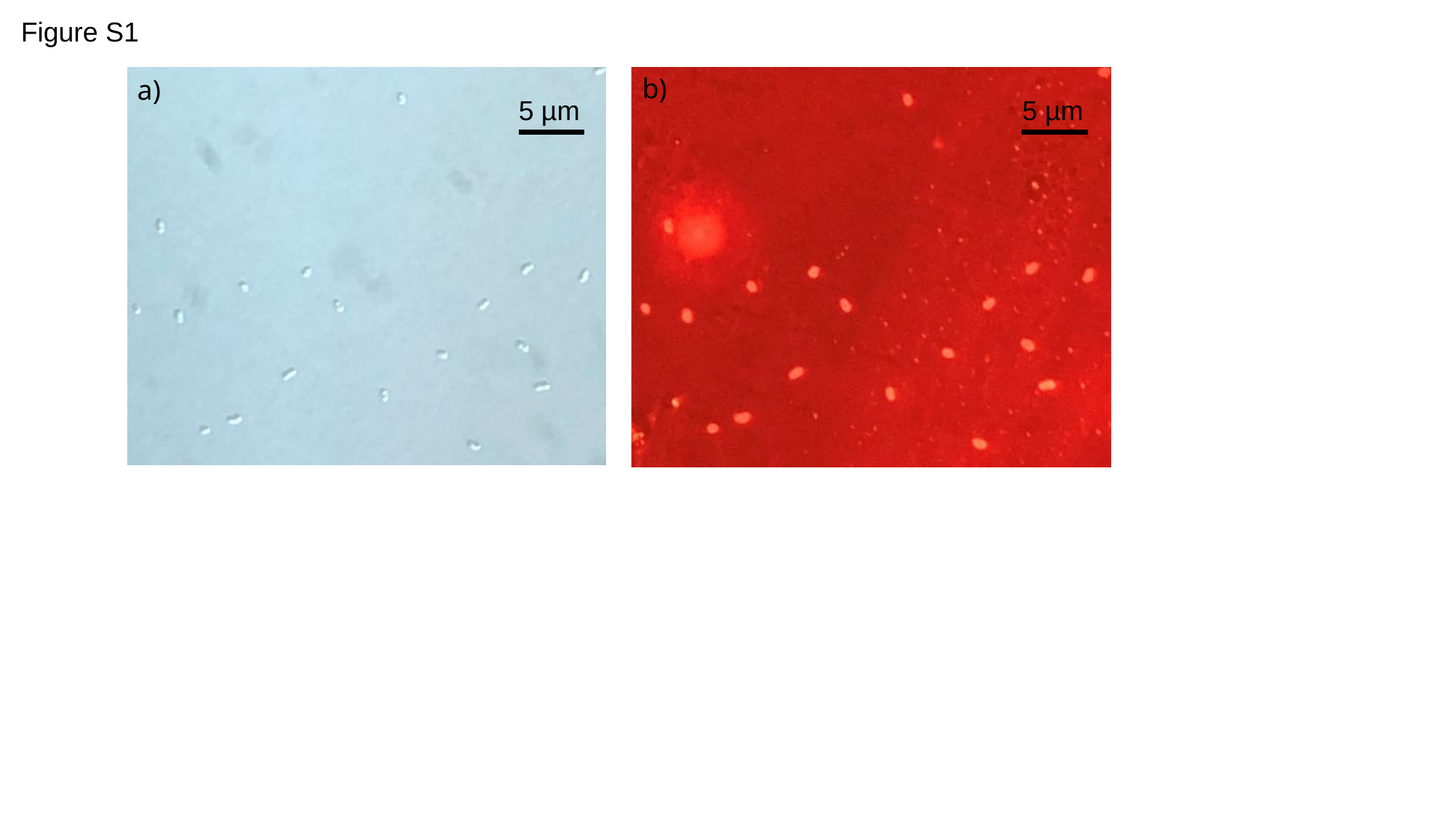

Figure S1
b)
a)
5 µm
5 µm

Supplement: Supplementary file 1 [file microorganisms-08-01719-s001.zip › 4.Supplementary_Figure_1.pptx]
